# Boosting Oxygen Reduction Reaction in Alkaline Media via Fe-N_x_-Enriched Carbon Electrocatalysts Derived from Waste Pod Shells

**DOI:** 10.3390/ma19214647

**Published:** 2026-10-31

**Authors:** Rui Yan, Kai Wang, Tao Zhang, Hewei Xu

**Affiliations:** 1 Department of Materials Engineering, Taiyuan Institute of Technology, Taiyuan 030008, China; ruiyan@tit.edu.cn (R.Y.); zhangt@tit.edu.cn (T.Z.); xuhewei@tit.edu.cn (H.X.); 2 Shanxi Center of Technology Innovation for Polyamide Materials, Taiyuan 030008, China

**Keywords:** biomass, pod shells, electrocatalysts, ORR, fuel cells

## Abstract

**Highlights:**

- A Fe/N-co-doped mesoporous carbon ORR catalyst was derived from biomass pod shells.
- A facile one-step pyrolysis route enabled sustainable catalyst synthesis.
- C-N-Fe-800 outperformed commercial Pt/C in durability and methanol tolerance.

**Abstract:**

A key challenge in advancing metal–air battery technologies is the development of low-cost oxygen reduction reaction (ORR) electrocatalysts with efficiency approaching that of premium benchmarks Pt/C. Herein, we report the synthesis of a Fe/N-co-doped carbon from a sustainable biomass pod shells, melamine, and iron acetate, employing a facile one-step pyrolysis approach. The optimized catalyst, C-N-Fe-800, exhibits good ORR activity in an alkaline medium, with an onset potential of 0.92 V and a half-wave potential of 0.79 V (vs. reversible hydrogen electrode (RHE)), surpassing the performance of commercial Pt/C in terms of durability and methanol tolerance. Good electrocatalytic performance stems from a synergistic contribution involving multiple active centers and structural merits. The active species Fe-N_x_ sites directly govern the intrinsic catalytic activity, while the presence of some mesopores, as indicated by the N_2_ adsorption–desorption isotherms, may aid electrolyte penetration and reactant transport, although the relatively low BET surface area limits the extent of this porosity. This work provides a strategic design principle for developing high-performance, non-precious-metal ORR catalysts from abundant biomass for application in alkaline energy conversion devices.

## 1. Introduction

Renewable energy technologies, notably fuel cells and water-splitting systems, have emerged as pivotal solutions to the pressing global challenges of energy scarcity and environmental degradation [1,2,3]. Within these systems, efficient catalysts for the oxygen reduction reaction (ORR) are essential. However, the reliance on platinum (Pt) to overcome the sluggish kinetics of the ORR remains the primary bottleneck to achieving cost-effective commercialization [4,5,6]. Consequently, the development of cost-effective alternatives with high activity and robust stability to replace precious metals has become a scientific and technological imperative [7,8,9].

Carbon materials are ideal candidates for electrochemical energy storage, conversion, and environmental protection, owing to their structural diversity, versatile surface chemistry, high controllability, and exceptional stability. Leveraging these advantages, the synergistic integration of transition metal oxides/nitrides within nitrogen-doped carbon matrices has emerged as a highly effective strategy for crafting high-performance electrocatalysts [10,11,12,13]. Among these, Fe-N-C materials have attracted particular attention because Fe-N_x_ moieties exhibit intrinsic activity toward the four-electron ORR pathway [14], making them the most promising non-precious-metal candidates [15].

Despite this promise, a key limitation persists in reported Fe-N_x_-based systems: conventional pyrolysis of Fe, N, and C precursors often leads to uncontrolled aggregation of iron species, burying a substantial fraction of Fe-N_x_ active sites within the carbon matrix or sealed pores, where they are inaccessible to reactants [16]. This issue is particularly relevant when biomass-derived precursors are used, since the intrinsic nitrogen content and the carbon architecture of the biomass are difficult to control, and the combined effect of the biomass carbon framework and an additional nitrogen source on the structure and ORR performance of Fe/N-co-doped carbons remains challenging to control [17]. These unresolved issues highlight the need for a simple route that couples a biomass-derived carbon framework with a supplementary nitrogen source to construct Fe/N-co-doped carbons for ORR catalysis.

Biomass resources, with their intrinsic sustainability, low cost, and global abundance, serve as an abundant and sustainable reservoir of carbon precursors, presenting a strategic alternative to tackle pressing energy and environmental issues [18,19,20]. Many biomass sources inherently contain elements such as oxygen, nitrogen, and potassium, which can function as dopants within a carbon matrix. The incorporation of these heteroatoms, which substitute for carbon atoms within the sp^2^ lattice of graphitic carbon, is a primary factor in conferring outstanding oxygen reduction reaction (ORR) performance. Consequently, a diverse range of biomass sources, including ginkgo biloba leaves [21], bagasse [22], and soybean [23], have been successfully utilized to produce carbon materials characterized by high specific surface areas and intricate natural architectures [24,25]. Nevertheless, the specific potential of pod shells for electrocatalyst synthesis remains unexplored, presenting a significant research opportunity. Pod shells, which exhibit a yellow lamellar structure and a relatively high nitrogen content, possess spontaneous biological characteristics that enable them to serve as both a precursor and a self-template for creating hollow carbon structures via a simple carbonization process. The resulting carbon material derived from pod shells features a mesoporous porous distribution and favorable conductive properties.

Herein, we address the above limitation by reporting a facile synthetic strategy to fabricate an Fe/N-co-doped carbon electrocatalyst (denoted as C-N-Fe-800). The synthesis employs pod shells as the carbon source and self-template, melamine as an additional nitrogen source to enrich the nitrogen content, and Fe(II) acetate as the iron precursor, involving a single-step calcination under a nitrogen atmosphere. The novelty of this approach lies in three aspects: (i) pod shells provide a lamellar, self-templating carbon framework, while the added melamine supplies supplementary nitrogen that promotes the formation of Fe-N_x_ moieties, enabling simultaneous control over carbon architecture and nitrogen doping in a single pyrolysis step; (ii) this single-step route simplifies the fabrication process compared with multi-step activation or template-removal procedures commonly required for porous Fe-N-C catalysts; and (iii) the resulting C-N-Fe-800 catalyst achieves a positive onset potential (E_0_ = 0.92 V vs. RHE) and half-wave potential (E_1/2_ = 0.79 V vs. RHE) via a dominant four-electron pathway in alkaline media, with good methanol tolerance and enhanced stability relative to commercial Pt/C. Consequently, the C-N-Fe-800 catalyst, derived from sustainable pod shells, presents a promising strategy for developing efficient and stable non-precious-metal electrocatalysts.

## 2. Materials and Methods

### 2.1. Materials

Pod shell powder (collected in Taiyuan city, China, and pulverized to powder in the laboratory), ethanol (C_2_H_4_OH, 99.7%, Sinopharm Chemical Reagent Co., Ltd., Shanghai, China), iron acetate (Fe(OH)(CH_3_COO)_2_, 99.0%, Maya Reagent, Mianyang, China), melamine (C_3_H_6_N_6_, ≥99.5%, Tianjin Guangfu Technology Development Co., Ltd., Tianjin, China), Pt/C (20 wt%, Shanghai Hesen Electric Co., Ltd., Shanghai, China) and Nafion solution (20 wt%, Shanghai Hesen Electric Co., Ltd., Shanghai, China) were all used as received without further treatment. Deionized water was applied for all experiments.

### 2.2. Synthesis of the Series of C-N-Fe Composites

A series of C-N-Fe composites were synthesized through a multi-step procedure. Initially, pod shell powder (1 g) and melamine (2 g) were dispersed in a mixed solvent of anhydrous ethanol (20 mL) and deionized water (10 mL) to form a suspension. Separately, an iron precursor solution was prepared by dissolving iron (II) acetate (0.014 g) in ethanol (25 mL). This precursor solution was then introduced into the pod shell suspension, and the resulting mixture was subjected to magnetic stirring at 600 rpm for 24 h. After the reaction, the product was collected and dried at 50 °C in a blast drying oven.

The dried material was subsequently pyrolyzed under a nitrogen atmosphere using a tube furnace. The pyrolysis process was carried out by maintaining the samples at designated temperatures of 600, 700, and 800 °C for 1 h, followed by natural cooling to room temperature. The final products were labeled as C-N-Fe-X, where X denotes the pyrolysis temperature (e.g., C-N-Fe-800). For comparison, two control samples were prepared under identical conditions: C-800, synthesized in the absence of both the nitrogen precursor and iron, and C-N-800, prepared without the addition of iron.

### 2.3. Material Characterization

A JSM-7200F scanning electron microscope (JEOL Ltd., Tokyo, Japan) was utilized to perform scanning electron microscopy (SEM) characterization. Transmission electron microscopy (TEM) images were acquired on a JEM-200F microscope (JEOL Ltd., Tokyo, Japan) operating at an acceleration voltage of 200 kV. X-ray powder diffraction (XRD) patterns were collected on a Panalytical Empyrean diffractometer (Malvern Panalytical, Almelo, The Netherlands) at a scanning rate of 0.02°/s with Cu Kα radiation (λ = 1.540600 Å). X-ray photoelectron spectroscopy (XPS) analysis was performed using a Thermo Scientific K-Alpha spectrometer (Thermo Fisher Scientific, Waltham, MA, USA; Al-Kα source, 500 μm spot). Raman spectroscopy was employed for further characterization on a HORIBA LabRAM HR Evolution system (HORIBA, Ltd., Kyoto, Japan) with a 532 nm excitation laser. Nitrogen physisorption measurements were performed using a Micromeritics ASAP 2460 system (Micromeritics Instrument Corp., Norcross, GA, USA), with the data analyzed to yield the pore-size distribution and Brunauer–Emmett–Teller (BET) specific surface area.

### 2.4. Electrochemical Measurements

A glassy carbon (GC) electrode (5 mm in diameter) was first polished with gamma-alumina powder, thoroughly rinsed with distilled water, and cleaned ultrasonically to remove any residues before being dried under vacuum.

For the catalyst ink preparation, 4 mg of the synthesized catalyst or 2 mg of commercial Pt/C was separately dispersed in a mixture of 1 mL ethanol and 10 μL of 5% Nafion solution via ultrasonication until a homogeneous suspension was obtained. A 20 μL aliquot of each ink was then drop-cast onto the polished GC surface and allowed to dry.

All electrochemical measurements were performed at room temperature using 0.1 M KOH solution as the electrolyte, which was saturated with O_2_ or N_2_ respectively. A platinum wire served as the counter electrode, and a saturated calomel electrode (SCE) was used as the reference electrode. All reported potentials were converted and are presented with respect to the reversible hydrogen electrode (RHE).

An Autolab electrochemical workstation (Metrohm Autolab, Utrecht, The Netherlands) coupled with an MSR electrode rotator (Pine Research Instrumentation, Durham, NC, USA) was employed for the measurements, during which cyclic voltammetry (CV) scans were carried out. Cyclic voltammetry was conducted with a scan rate of 50 mV s^−1^. Linear sweep voltammetry (LSV) was conducted at 10 mV s^−1^ with 1600 rpm rotation speed. Prior to each ORR test, the electrolyte was saturated with O_2_ via 30 min purging. The H_2_O_2_ yield and electron-transfer number (n) were determined, using the rotating ring-disk electrode (RRDE) technique. A rotating ring-disk electrode (RRDE, SD = 0.247 cm^2^) served as the bare working electrodes. The peroxide yield (H_2_O_2_%) and the electron-transfer number (n) were determined through the following equations [26]:

H_2_O_2_% = 200 × (I_r_/N)/(I_d_ + I_r_/N) (1)

n = 4 × I_d_/(I_d_ + I_r_/N) (2)

where I_d_ and I_r_ are the disk and ring currents, respectively. N is the current collection efficiency of the Pt ring (0.37). The ring potential is kept at a potential of 1.2 V/RHE.

## 3. Results and Discussion

**Figure 1:**
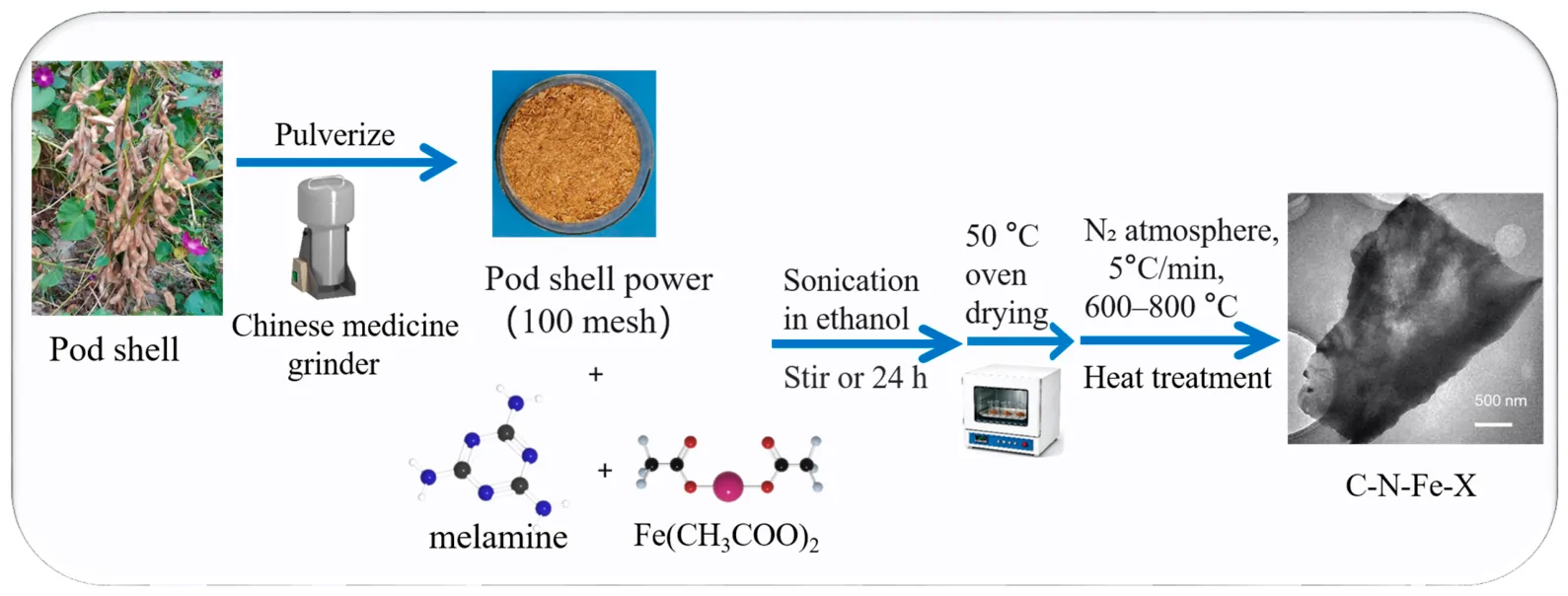
Synthesis procedure of C-N-Fe-X.

Figure 1 illustrates the synthesis procedure for the C-N-Fe-X composite. The morphological characteristics of the electrocatalysts, prepared under varying conditions, were analyzed using SEM and TEM, respectively.

**Figure 2:**
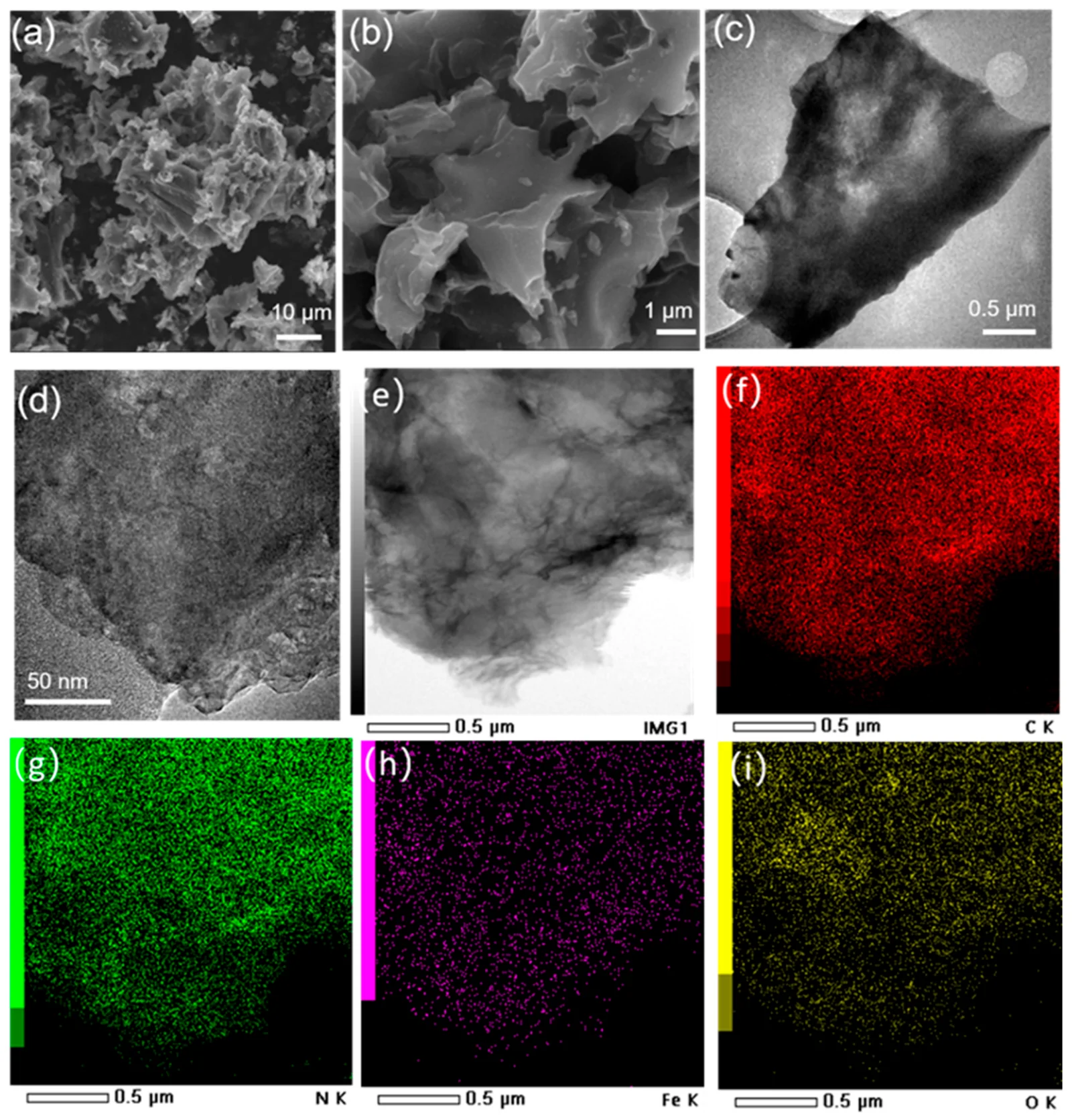
SEM images of (**a**) C-800, (**b**) C-N-800, (**c**,**d**) TEM images of C-N-Fe-800 at different magnification, (**e**) STEM Image, elemental mapping of (**f**) C, (**g**) N, (**h**) Fe, and (**i**) O elements of C-N-Fe-800.

As shown in Figure 2a, the C-800 sample exhibited a porous sheet-like morphology, while C-N-800 and C-N-Fe-800 (Figure 2b and Figure S1) also displayed a similar lamellar structure. TEM analysis of the C-N-Fe-800 sample (Figure 2c,d) reveals a layered morphology consisting of a carbon base layer derived from pod shells overlaid with a lamellar carbon structure. To elucidate the spatial distribution of the constituent elements, scanning transmission electron microscopy (STEM) combined with energy-dispersive X-ray spectroscopy (EDS) mapping was performed (Figure 2e–i). The C, N, Fe and O signals are all uniformly distributed throughout the selected carbon sheet. In particular, the Fe mapping (Figure 2h) appears as finely dispersed bright dots without any localized enrichment, corroborating the absence of Fe agglomerates observed by TEM. The homogeneous distribution of N further indicates that N atoms are successfully incorporated into the carbon lattice and may serve as anchoring sites for isolated Fe centers (i.e., Fe–N_x_ moieties). Collectively, these results demonstrate that C-N-Fe-800 consists of a layered, N-doped carbon sheet decorated with highly dispersed Fe sites, a configuration that is expected to offer abundant accessible active sites together with efficient electron and mass transport for oxygen reduction reaction.

**Figure 3:**
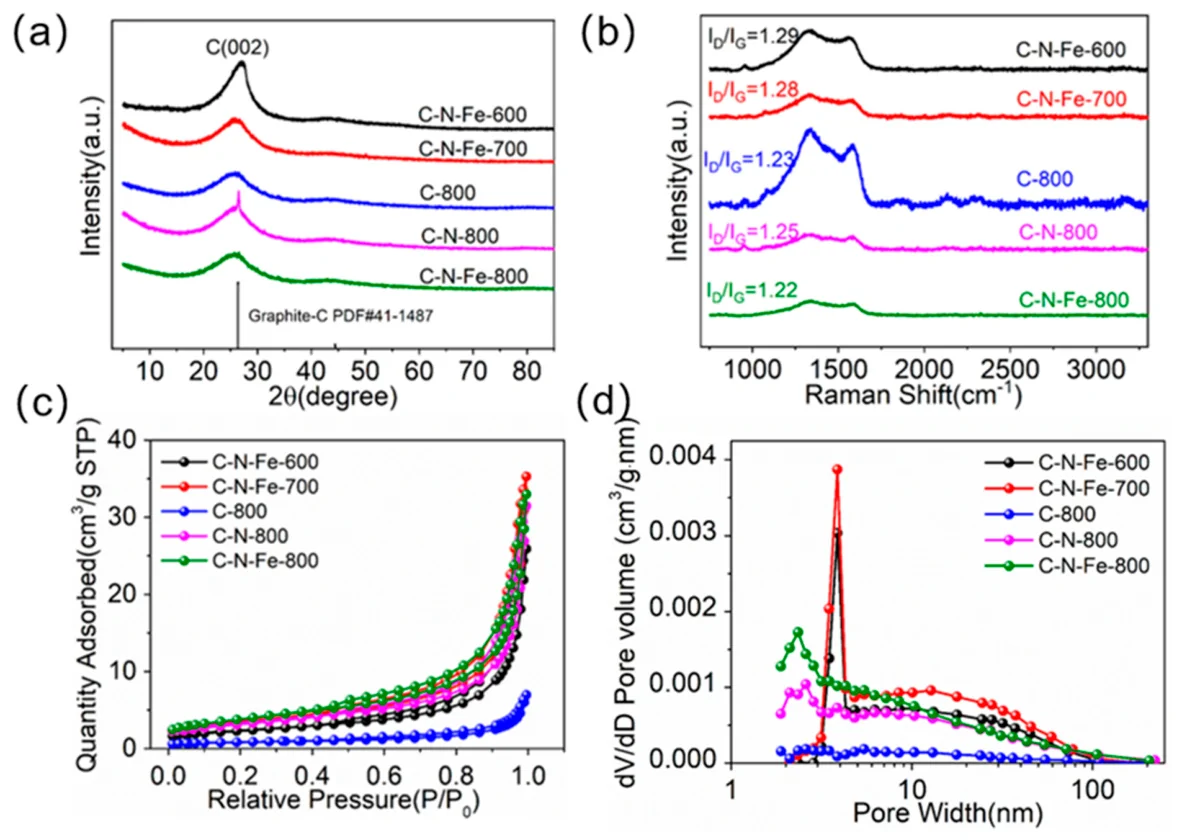
(**a**) XRD patterns, (**b**) Raman spectra, (**c**) N_2_ adsorption and desorption isotherms, and (**d**) pore-size distribution of the samples.

Figure 3a presents the XRD patterns used to characterize the crystalline phases of the synthesized electrocatalysts. A broad peak centered at approximately 26°, corresponding to the (002) plane of graphitic carbon (PDF#41–1487), is observed in all patterns, indicating the presence of a largely amorphous carbon matrix. A sharp peak at 26° is also observed for C-N-800 implying the coexistence of a disordered, essentially amorphous carbon component alongside the partially graphitic carbon phase. Notably, no diffraction peaks attributable to metallic Fe, iron carbides, or iron oxides are detected across the entire scanned range, suggesting that any Fe-containing species in these materials are dispersed as atomically distributed Fe-N_x_ sites within the carbon matrix.

Raman spectroscopy was employed to characterize the structural evolution and defect density of the graphitic carbon matrices (Figure 3b). All catalysts exhibit two characteristic bands: the D band at approximately 1343 cm^−1^, indicative of structural defects and disordered carbon, and the G band at 1560 cm^−1^, corresponding to the in-plane vibration of sp^2^-hybridized graphitic carbon. The relative intensity ratio of these bands (I_D_/I_G_) serves as a quantitative measure of the defect density [27,28]. As shown in Figure 3b, the I_D_/I_G_ ratio decreases systematically with increasing pyrolysis temperature. This trend signifies a reduction in the defect concentration of the carbon atomic lattice and an enhancement in graphitic ordering, attributable to the structural reorganization during high-temperature treatment. Concurrently, the peak shifts and attenuated intensity observed at elevated temperatures can be attributed to a decrease in particle size and pore aperture. Among the series, the C-N-Fe-800 composite exhibits the lowest I_D_/I_G_ value, denoting its highest degree of graphitization. Among the samples, C-N-Fe-800 exhibits an intermediate I_D_/I_G_ value of ~1.22. Beyond this point, a further increase in I_D_/I_G_ (excessive disorder) leads to a decline in E_1/2_, likely due to compromised electrical conductivity and/or overly strong intermediate adsorption. The graphitic structure is conducive to the oxygen reduction reaction (ORR) by improving electrical conductivity and facilitating electron transfer, thereby accelerating reaction kinetics [29,30].

The porous texture of the C-N-Fe-X samples were probed by N_2_ adsorption–desorption measurements (Figure 3c,d). The pore structure properties of the C-N-Fe-X materials were characterized by nitrogen physisorption measurements. Figure 3 presents the corresponding N_2_ adsorption–desorption isotherms. At low relative pressures, almost no gas adsorption was observed, indicating a negligible amount of micropores. The adsorption isotherms reached equilibrium rapidly and exhibited a distinct hysteresis loop with typical Type-IV behavior, confirming the presence of a mesoporous structure. For the sample C-N-Fe-800, the adsorption capacity increased markedly at higher relative pressures, suggesting the coexistence of mesopores and macropores. The pore-size distributions derived from the desorption branch using the Barrett–Joyner–Halenda (BJH) method are shown in Figure 3d. The majority of pores fall within the range of 2–100 nm. The specific surface areas of C-N-Fe-600, C-N-700, C-800, C-N-800, and C-N-Fe-800 calculated by the BET method are 8.4, 12.4, 3.0, 12.2 and 13.5 m^2^ g^−1^, respectively. Furthermore, pore-volume analysis reveals that mesopores account for over 90% of the total pore volume, demonstrating that the porosity of these materials is predominantly mesoporous. The presence of some mesopores, as indicated by the N_2_ adsorption–desorption isotherms, may facilitate electrolyte penetration and reactant transport to a certain extent; however, given the relatively low BET surface area (13.5 m^2^ g^−1^), the porosity should be regarded as limited rather than well-developed, and the number of accessible active sites is therefore not expected to be primarily governed by mesoporosity [30,31].

**Figure 4:**
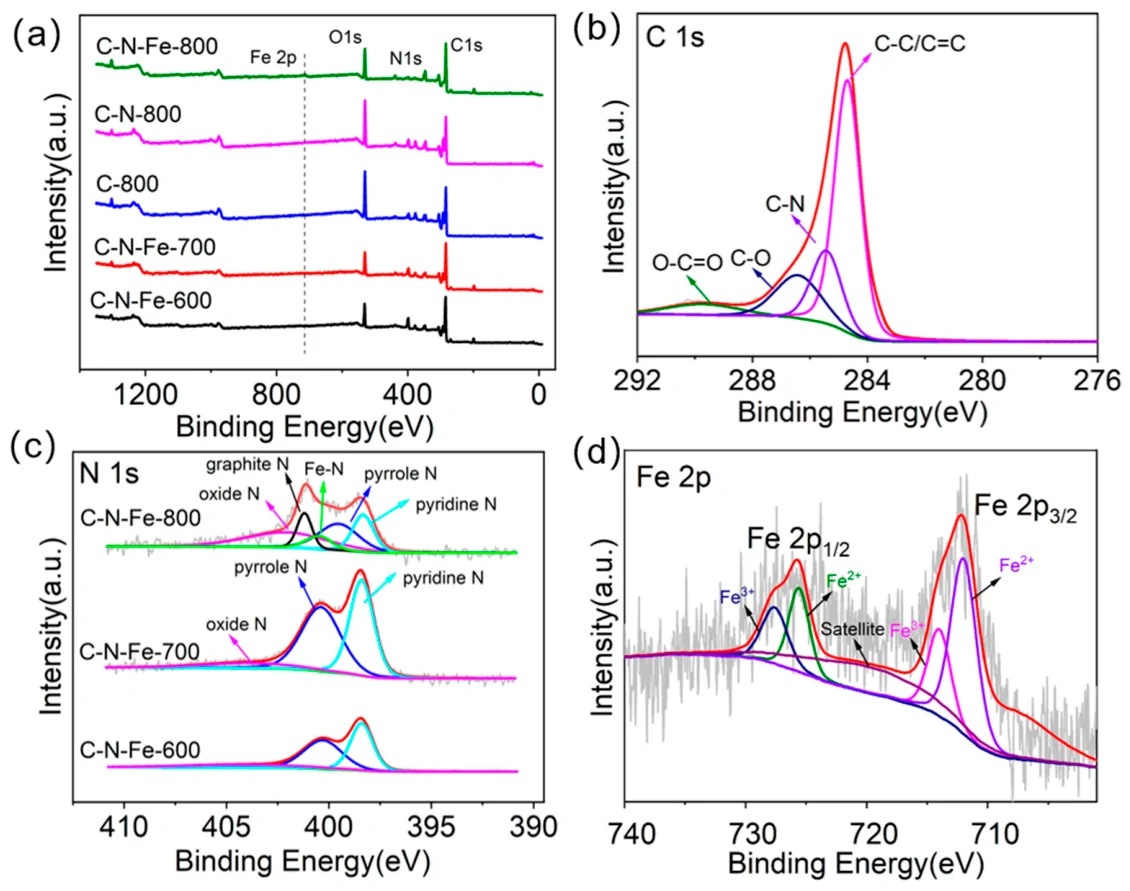
(**a**) XPS survey spectra, (**b**) C1s of C-N-Fe-800, (**c**) N1s of C-N-Fe-X, and (**d**) Fe 2p of C-N-Fe-800.

The elemental composition of the surface and chemical states of the electrocatalysts were investigated using XPS analysis. The survey spectrum of the C-N-Fe-800 catalyst (Figure 4a) confirms the presence of carbon (71.11 at%), oxygen (22.54 at%), nitrogen (5.34 at%), and iron (1.01 at%). The high-resolution C 1s spectrum (Figure 4b) was deconvoluted into four constituent peaks at binding energies of 284.7, 285.4, 286.5 and 290.0 eV, which are attributed to the C=C/C-C, C-N, C-O and O-C=O bonds, respectively [32,33,34,35]. The presence of oxygen-containing functional groups is correlated with enhanced surface wettability. This is substantiated by contact angle measurements (Figure S3), where the value for C-N-Fe-800 (18.3°) is significantly lower than that of C-800 (32.7°). This indicates that nitrogen and oxygen co-doping markedly improves hydrophilicity, thereby ensuring the active sites can be more readily accessible to the aqueous electrolyte [36,37]. To further examine the origin of the improved surface wettability, the C–O and O–C=O fractions were quantified from the high-resolution C 1s XPS spectra for all samples (Figure S2 and Table S1). The contents of these oxygen-containing groups follow the order C-N-Fe-800 > C-N-800 > C-N-Fe-700, which is consistent with the wettability trend observed for these samples, supporting the view that oxygen-containing functional groups contribute to enhanced surface wettability.

Further investigation of the high-resolution N1s spectra was conducted on each N-doped sample, as illustrated in Figure 4c and Figure S4. As shown in Table 1, the N contents of C-N-Fe-600, C-N-Fe-700, C-800, C-N-800 and C-N-Fe-800 were estimated to be 14.87 at%, 11.68 at%, 7.76 at%,9.96 at% and 5.34 at%, respectively, indicating that C-800 has a very high nitrogen content (7.76 at%), and adding melamine resulted in higher N doping (9.96 at%). A clear decreasing trend in total N content is observed with increasing pyrolysis temperature for the C-N-Fe series. Nitrogen-containing functional groups exhibit varying thermal stabilities. As the pyrolysis temperature increases (from 600 to 800 °C), less stable N species (e.g., pyrrolyl N, pyridinic N, and especially oxidized N) undergo thermal decomposition and volatilization, leading to a significant loss of total N. This is a well-established phenomenon in carbon material synthesis.

The presence of Fe during pyrolysis can catalyze the graphitization of the carbon matrix. While this often improves electrical conductivity, the associated restructuring and ordering of carbon layers at high temperatures can simultaneously promote the removal of nitrogen atoms from the lattice, as they may be expelled during the formation of more stable graphitic domains. This catalytic loss mechanism is a primary reason for the lower total N in C-N-Fe-800.

The high-resolution spectrum of the fitted N 1s region for C-N-Fe-800 can be divided into five peaks assigned to pyridinic N (398.3 eV), pyrrolic N (400.0 eV), graphitic N (401.2 eV), Fe-N (400.5 eV) and oxidized N (402.8 eV) [38,39]. The high-resolution spectrum of the fitted N 1s region for C-800 and C-N-800 can be divided into three peaks belonging to pyridinic N (398.3 eV), pyrrolic N (400.0 eV), graphitic N (401.2 eV) (Figure S4). In addition, the XPS results show that the peaks located at 401.2 eV have disappeared in C-N-Fe-600 and C-N-Fe-700, respectively. This result confirms the successful incorporation of nitrogen species into the carbon matrix. Notably, the peak at 400.5 eV confirms the presence of Fe-N_x_ bonds, indicating the generation of Fe-N_x_ active sites [40,41].

The iron content was quantified by XPS. Based on the XPS survey spectra, the atomic Fe content is 0.57 at% for C-N-Fe-600, 0.59 at% for C-N-Fe-700, and 1.01 at% for C-N-Fe-800 (Table S2). The high-resolution Fe 2p spectrum (Figure 4d) was fitted with six distinct peaks. The doublet located at 712.0 eV and 725.6 eV is assigned to Fe^2+^ species (2p_3/2_ and 2p_1/2_, respectively), whereas the doublet observed at 714.1 eV and 727.7 eV is attributed to Fe^3+^ species. A common interpretation of XPS data supports the presence of Fe-N_4_-C structures in the catalysts, consistent with prior reports. This can be regarded as primary evidence. The coordination environment of iron with nitrogen waits for clarification. Techniques capable of providing this information, including FT-EXAFS and HAADF-STEM, have not been employed in the current study; we refer to the sites here as Fe-N_x_-C, indicating that further verification is needed [42].

**Figure 5:**
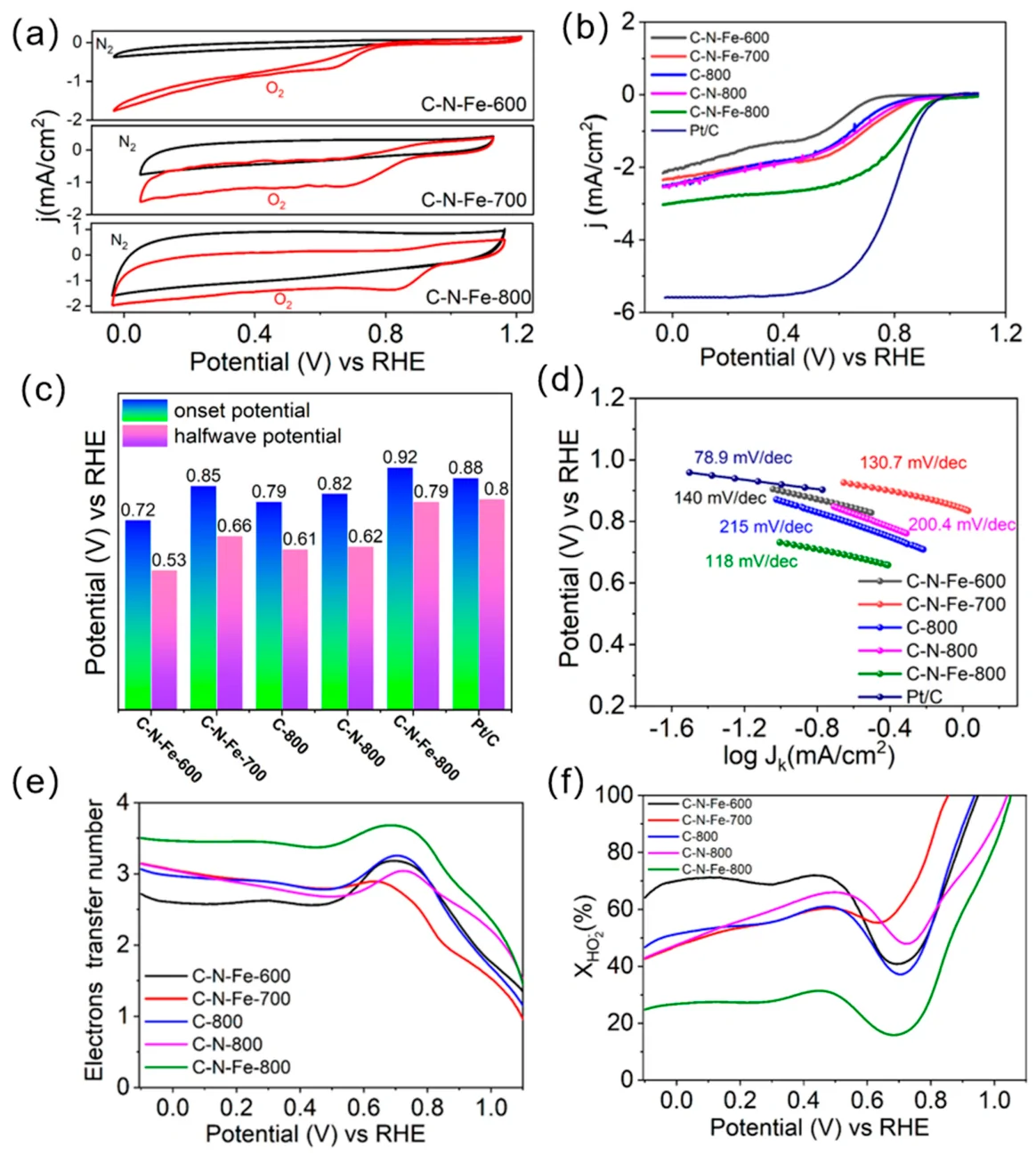
(**a**) CV curves measured for the prepared electrocatalysts in both N_2_- and O_2_-saturated 0.1 M KOH electrolyte solution at a scan rate of 50 mV s^−1^, (**b**) LSV curves of the electrocatalysts measured at 10 mV s^−1^ and 1600 rpm in O_2_-saturated 0.1 M KOH, (**c**) comparison of the onset potential and half-wave potential for all electrocatalysts, (**d**) Tafel plots of all electrocatalysts, (**e**) electron-transfer number (n) at different potential, and (**f**) H_2_O_2_ production yields at different potential.

The electrocatalytic performances of the synthesized electrocatalysts for the oxygen reduction reaction (ORR) were evaluated and summarized in Figure 5, Figure S5 and Figure S6. Cyclic voltammetry (CV) measurements conducted in an N_2_-saturated 0.1 M KOH electrolyte produced featureless curves for all the electrocatalysts. In O_2_-saturated electrolyte, however, well-defined cathodic reduction peaks were observed, with peak potentials centered at 0.61 V, 0.64 V and 0.81 V (vs. RHE) for C-N-Fe-600, C-N-Fe-700, and C-N-Fe-800, respectively. These results confirm the intrinsic ORR catalytic activity of the materials [43,44].

Linear sweep voltammetry (LSV) was carried out with a rotating disk electrode (RDE) at 1600 rpm in O_2_-saturated 0.1 M KOH (Figure 5b). The C-N-Fe-800 catalyst exhibited an onset potential (E_onset_) of 0.92 V and a half-wave potential (E_1/2_) of 0.79 V, values comparable to those of a commercial Pt/C catalyst (E_onset_ = 0.88 V, E_1/2_ = 0.80 V) (Figure 5c). Tafel analysis was conducted to investigate the oxygen reduction reaction kinetics. The Tafel slope (Figure 5d) is converted from the linear sweep voltammetry curve (Figure 5b). Among the series, the C-N-Fe-800 catalyst exhibited the smallest Tafel slope (118 mV dec^−1^), indicating the most favorable ORR kinetics. This value falls within the range reported for Fe-N-C catalysts in alkaline media, where Tafel slopes ranging from ~50 to ~120 mV dec^−1^ have been documented depending on the active site structure and test conditions [45,46,47].

The electron-transfer pathway of the oxygen reduction reaction (ORR) was further investigated using a rotating ring-disk electrode (RRDE). As shown in Figure 5e,f, the C-N-Fe-800 catalyst exhibits a relatively low H_2_O_2_ yield (∼15%) over the scanned potential range. This result suggests that the ORR over C-N-Fe-800 proceeds primarily through a dominant 4e^−^ pathway, accompanied by a minor 2e^−^ contribution. The other samples undergo an ORR catalytic process dominated by 2e^−^. Moreover, C-N-Fe-800 demonstrated superior methanol tolerance and better cycling stability compared to the Pt/C benchmark (Figure S6b,c).

To evaluate whether melamine acts solely as a nitrogen source or also as a porogene, a melamine-free control (iron acetate and pod shells only, pyrolyzed at 600–800 °C) was prepared and compared electrochemically under identical conditions (Figure S7). The melamine-free control shows no discernible reduction peak in CV, whereas the melamine-containing samples exhibit a clear cathodic peak, indicating a higher density of electrochemically accessible active sites in the latter, consistent with their higher ORR activity. However, because BET and XPS characterization of the control was not performed, the nitrogen-doping and porogene contributions of melamine cannot be quantitatively separated in this work.

To further identify the active sites, we conducted SCN^−^ poisoning and acid-etching experiments of C-N-Fe-800 (Figure S8) [48]. The ORR activity (half-wave potential) of C-N-Fe-800 was significantly suppressed after SCN^−^ treatment, confirming that iron-based species (Fe-N_x_-C) are active centers. Mild acid washing (to remove exposed/less stable iron particles) led to a partial but not complete loss of activity. The residual activity strongly suggests the contribution of acid-resistant active sites, which could include stable Fe-N_x_ embedded within the carbon matrix.

An integrated assessment of the extensive RDE and stability tests reveals that the mesoporosity primarily enhances mass transport and active site accessibility, while an increased degree of graphitization mainly improves electrical conductivity. Specific nitrogen configurations, such as Fe-N_x_ and graphitic N, are directly associated with the modulation of intrinsic catalytic activity. The optimal performance of C-N-Fe-800 does not result from extreme values in any single parameter, but rather from a synergistic balance achieved under specific synthesis conditions (800 °C).

To directly assess the electrochemically accessible surface area (ECSA), the double-layer capacitance (C_dl_) was measured in the non-faradaic potential window at different scan rates by CV, and ECSA was calculated as ECSA = C_dl_/C_s_ with C_s_ = 0.040 mF cm^−2^ (Figure S9). The ECSA values follow the order C-N-Fe-800 > C-N-800 > C-N-Fe-700 > C-800 > C-N-Fe-600 (20.48, 16.27, 10.34, 6.52, and 1.81 cm^2^, respectively). This trend is consistent with the observed electrochemical performance and indicates that a larger electrochemically accessible area contributes to higher ORR activity. Given the relatively low BET surface areas of these samples, the ECSA difference is more likely related to the density of electrochemically accessible Fe-N_x_ sites than to the extent of porosity alone.

The RRDE and RDE results suggest that the C-N-Fe-800 catalyst demonstrates promising potential as an alternative to Pt/C for the oxygen reduction reaction. For comparison, ORR performance data of catalysts derived from various other biomass sources reported in the literature are summarized in Table S3. The C-N-Fe-800 catalyst prepared from pod shell within this work, while not achieving the highest ORR activity among all biomass-derived catalysts, offers superior advantages in terms of its simple preparation process and low cost.

## 4. Conclusions

In summary, a series of high-performance oxygen reduction reaction (ORR) electrocatalysts were successfully synthesized through a straightforward one-step pyrolysis of sustainable pod shell biomass, melamine, and iron acetate. The optimized C-N-Fe-800 composite exhibits good ORR activity in an alkaline medium, rivaling that of commercial Pt/C. This performance originates from a synergistic combination of active centers and structural advantages: Fe-N_x_-C active sites contribute directly to the intrinsic catalytic activity, while the presence of some mesopores, as indicated by the N_2_ adsorption–desorption isotherms, may aid electrolyte penetration and reactant transport. Additionally, an increased degree of graphitization enhances electrical conductivity throughout the material. Overall, this work provides a practical and sustainable strategy for designing efficient non-precious-metal electrocatalysts from biomass for use in alkaline-based energy conversion technologies.

## Figures and Tables

**Table 1:** Relative atomic percentage of various nitrogen species in the electrocatalysts, derived from the XPS N1s peak fitting results.

Catalyst	Nitrogen Atomic Ratio (%)	Pyridine N (%)	Pyrrolyl N (%)	Graphitic N (%)	Oxidized N (%)	Fe-N_x_ (%)
C-N-Fe-600	14.87	6.33	6.46	—	2.08	—
C-N-Fe-700	11.68	5.13	5.48	—	1.07	—
C-800	7.76	3.22	1.78	2.76	—	—
C-N-800	9.96	4.26	3.33	2.37	—	—
C-N-Fe-800	5.34	1.06	1.43	0.70	1.68	0.46

## Data Availability

The original contributions presented in this study are included in the article/Supplementary Materials. Further inquiries can be directed to the corresponding author.

## Supplementary Materials

The following supporting information can be downloaded at https://www.mdpi.com/article/10.3390/ma19214647/s1, Figure S1: The SEM image of C-N-Fe-800; Figure S2: XPS survey spectra C1s of (a) C-N-Fe-600, (b) C-N-Fe-700, (c) C-800 and (d) C-N-800; Figure S3: Contact angle of (a) C-N-Fe-600, (b) C-N-Fe-700, (c) C-800, (d) C-N-800, and (e) C-N-Fe-800; Figure S4:XPS survey spectra N1s of C-800 and C-N-800; Figure S5: (a) Cyclic voltammetry curve of C-800, (b) cyclic voltammetry curve of C-N-800, Figure S6: (a) Electron-transfer numbers of all samples, (b) anti-methanol poisoning performance of C-N-Fe-800 and Pt/C, and (c) cycle stability performance of C-N-Fe-800 and Pt/C; Figure S7: (a–c) CV curves of as prepared electrocatalysts in N_2_ and O_2_-saturated 0.1 M KOH at a scan rate of 50 mV s^−1^, (d) LSV curves of as prepared electrocatalysts in O_2_-saturated 0.1 M KOH at a scan rate of 10 mV s^−1^, rotation rate =1600 rpm. Figure S8: (a) LSV curves of C-N-Fe-800 before and after in 0.1 M KOH+10 mM KSCN, (b) LSV curves of C-N-Fe-800 before and after acid-etching in 0.1 M KOH; Figure S9: Electrochemical double-layer capacitance (C_dl_) characterization for ECSA calculation. (a–e) Cyclic voltammograms of catalysts at scan rates of 10–60 mV s^1^ in a non-Faradaic potential range of 0.92–1.02 V vs. RHE. (f) linear plots of Δj/2 versus scan rate, yielding C_dl_ values for catalysts, respectively. Table S1: Relative percentage of O-C=O/C-O in the electrocatalysts, derived from the XPS C 1s; Table S2: Relative atomic percentage of Fe in the electrocatalysts, derived from the XPS Fe 2p;Table S3: Comparison of half-wave potentials for ORR of biomass-based catalysts from the literature and this work in alkaline media. Refs. [49,50,51,52,53,54,55,56,57,58,59] are cited in Supplementary materials file.
