# Supplementary material for: Boosting Oxygen Reduction Reaction in Alkaline Media via Fe-Nx-Enriched Carbon Electrocatalysts Derived from Waste Pod Shells
Source: Materials (Basel). 2026 Oct 31;19(21):4647. doi: 10.3390/ma19214647 (PMC13764710; doi:10.3390/ma19214647)
Supplement: Supplementary file 1 [file materials-19-04647-s001.zip › materials-4619223-supplementary.pdf]

Supporting Information

Boosting Oxygen Reduction Reaction in Alkaline Media via Fe−N_x-Enriched Carbon Electrocatalysts Derived from Waste Pod Shells

Rui Yan[a], Kai Wang[a,b]*, Tao Zhang[a], Hewei Xu[a]

[a]Department of Materials Engineering, Taiyuan Institute of Technology, Taiyuan 030008, China

[b]Shanxi Center of Technology Innovation for Polyamide Materials, Taiyuan 030008, China

Electrochemically active surface area (ECSA): The double-layer capacitance (C_{dl}) was determined for the catalysts to determine ECSA. In order to get C_{dl}, a non-Faradaic potential region (0.92-1.02 V vs RHE) where there is no redox process taking place must be identified from the CV curve obtained in N_2-saturated 0.1 M aqueous solution of KOH. Afterwards, scan rate dependent CVs were obtained basically in a 0.1 V window around the open circuit voltage. The various scan rates are: 10, 20, 30, 40, 50, 60 mV s^{-1}. The electrochemical active surface area (ECSA) was calculated based on the double-layer capacitance (C_{dl}) using the following equation:

$$ECSA= C_{dl}/Cs \quad (S1)$$

where:

- C_{dl} = double-layer capacitance obtained from the slope of Δj/2 vs. scan rate plot (mF cm^{-2})
- C_s = specific capacitance of a flat surface (typically 0.040 mF cm^{-2}, a widely adopted moderate value for ECSA estimation) [49-50]

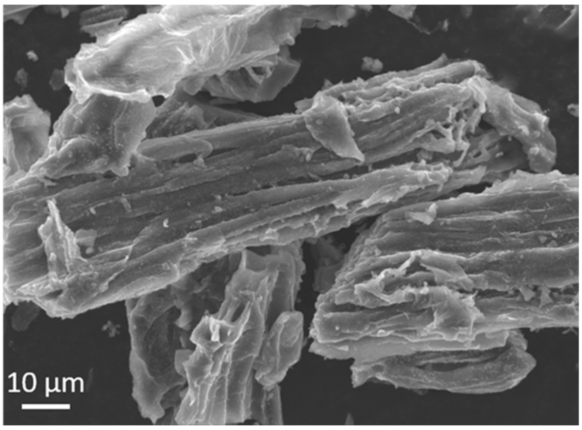

Figure S1. The SEM image of C-N-Fe-800

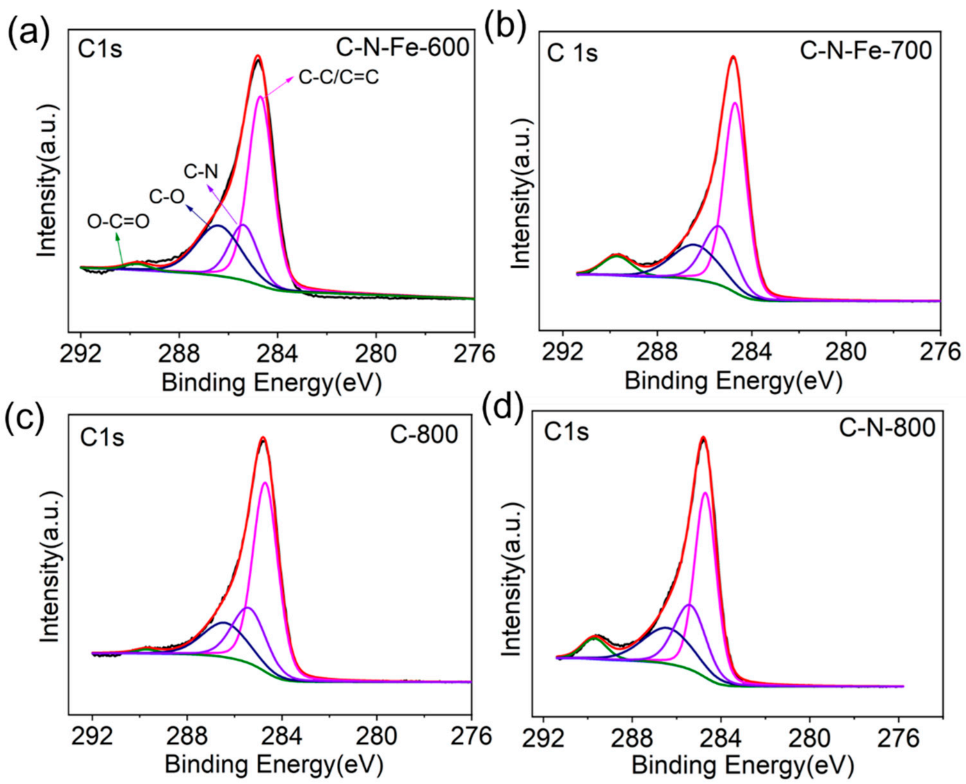

Figure S2.XPS survey spectra C1s of (a) C-N-Fe-600, (b) C-N-Fe-700, (c) C-800 and (d) C-N-800.

Table S1 Relative percentage of O-C=O/C-O in the electrocatalysts, derived from the XPS C 1s

**Catalyst**	**O-C=O/C-O percentage**
C-N-Fe-600	22 %
C-N-Fe-700	26 %
C-800	20 %
C-N-800	27.5 %
C-N-Fe-800	28 %

Table S2 Relative atomic percentage of Fe in the electrocatalysts, derived from the XPS Fe2p

**Catalyst**	**Fe atomic ratio (%)**
C-N-Fe-600	0.57
C-N-Fe-700	0.59
C-N-Fe-800	1.01

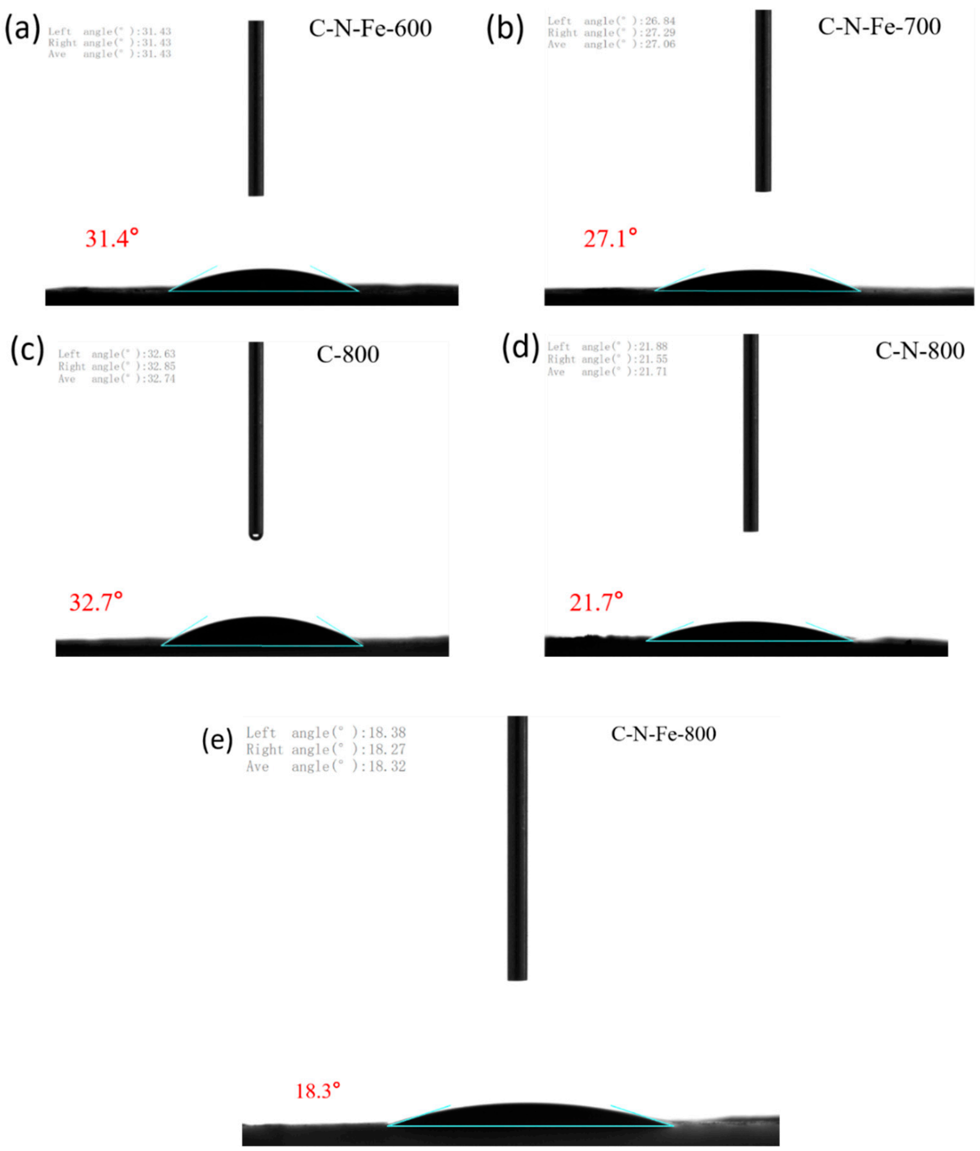

Figure S3. Contact angle of (a) C-N-Fe-600, (b) C-N-Fe-700, (c) C-800, (d) C-N-800, (e) C-N-Fe-8

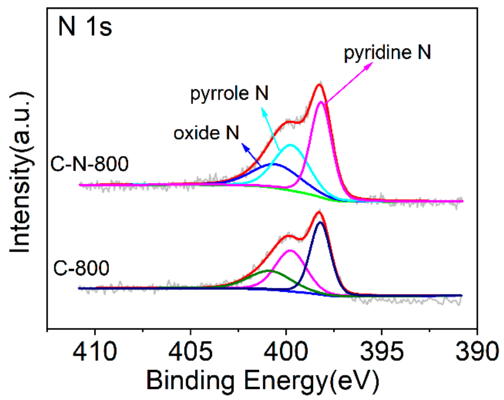

Figure S4. XPS survey spectra N1s of C-800 and C-N-800.

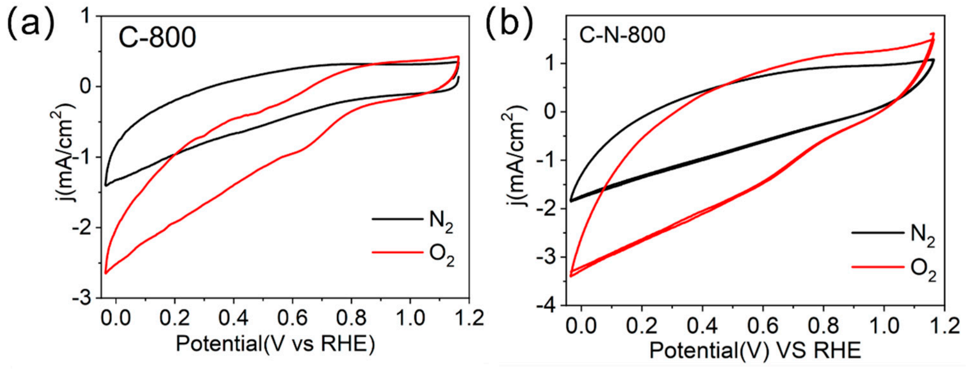

Figure S5. (a) Cyclic voltammetry curve of C-800, (b) Cyclic voltammetry curve of C-N-800.

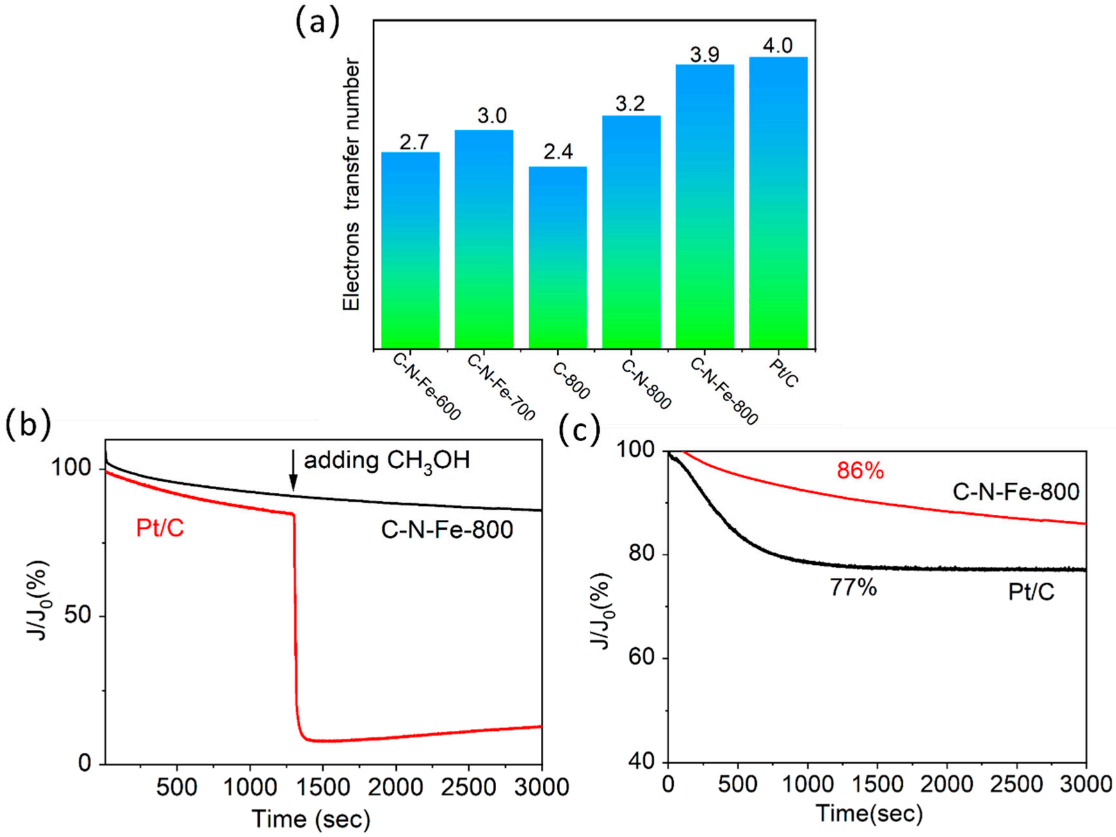

Figure S6. (a) Electron transfer numbers of all samples, (b) Anti-methanol poisoning performance of C-N-Fe-800 and Pt/C, (c) Cycle stability performance of C-N-Fe-800 and Pt/C.

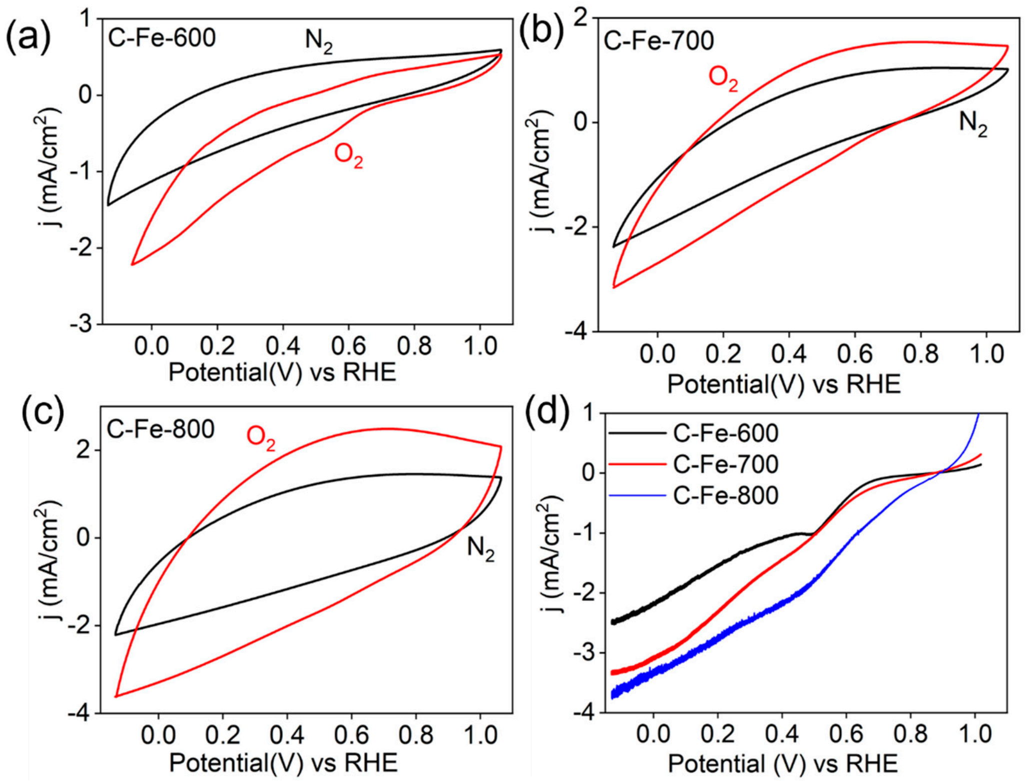

Figure S7. (a-c) CV curves of as prepared electrocatalysts in N_2 and O_2-saturated 0.1 M KOH at a scan rate of 50 mV s^{-1}, (d) LSV curves of as prepared electrocatalysts in O_2-saturated 0.1 M KOH at a scan rate of 10 mV s^{-1}, rotation rate =1600 rpm.

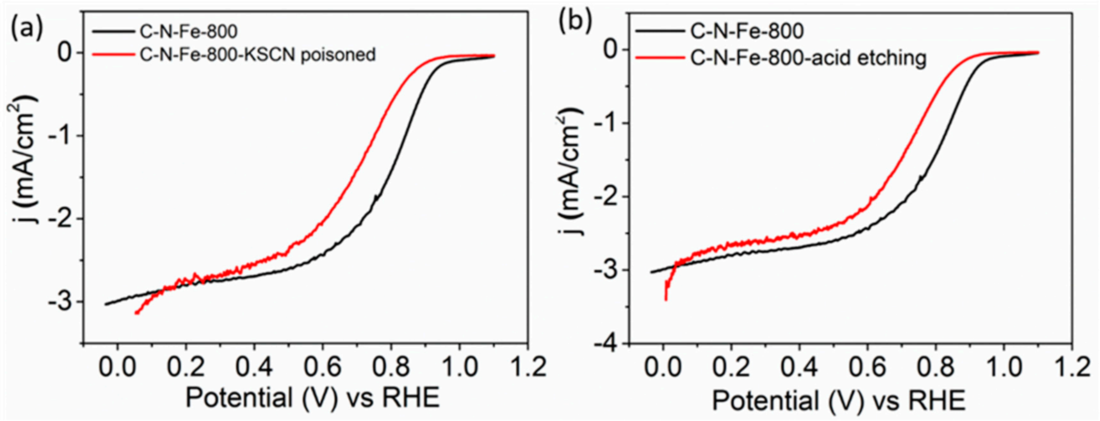

Figure S8. (a) LSV curves of C-N-Fe-800 before and after in 0.1 M KOH+10 mM KSCN, (b) LSV curves of C-N-Fe-800 before and after acid-etching in 0.1 M KOH

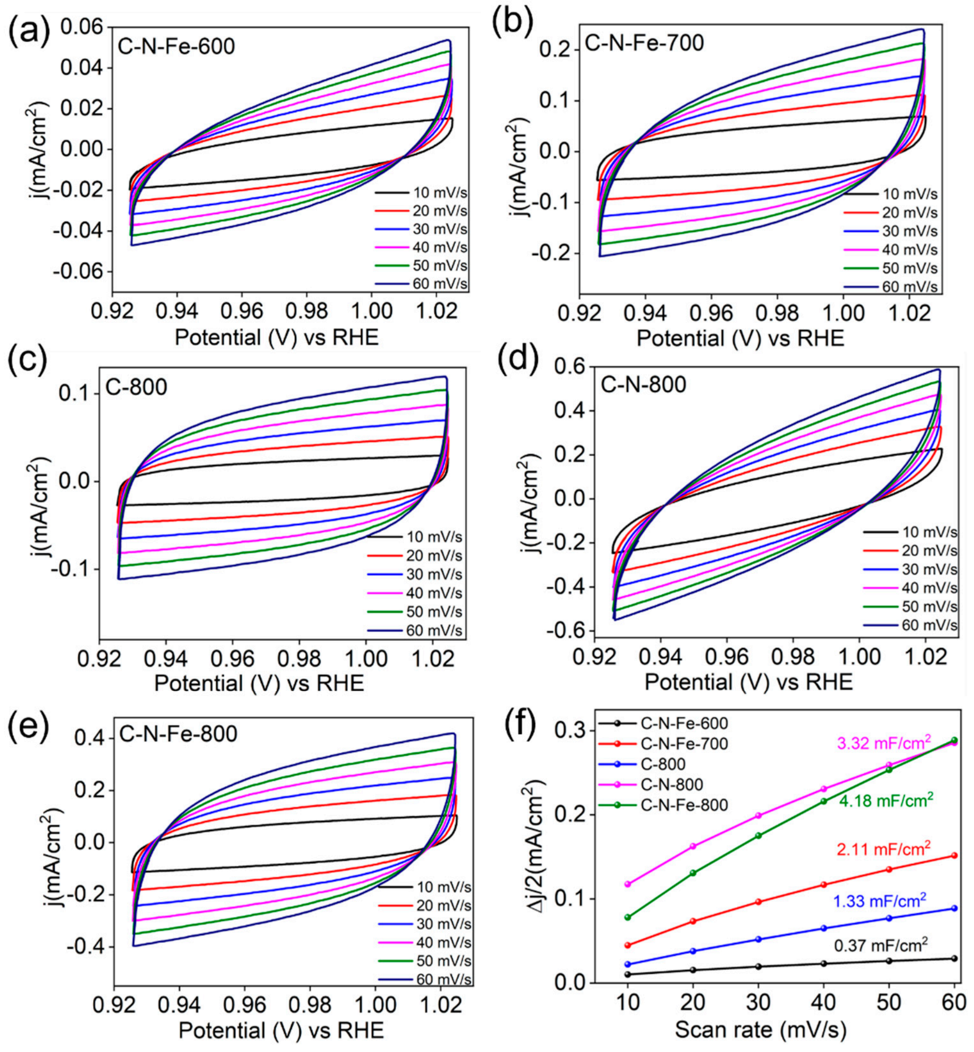

Figure S9 Electrochemical double-layer capacitance (C_{dl}) characterization for ECSA calculation. (a-e) Cyclic voltammograms of catalysts at scan rates of 10-60 mV s^{-1} in a non-Faradaic potential range of 0.92-1.02 V vs. RHE. (f) Linear plots of $\Delta j/2$ versus scan rate, yielding C_{dl} values for catalysts, respectively.

Table S3. Comparison of half-wave potentials for ORR of biomass-based catalysts from literature and this work in alkaline media

Catalyst	Electrolyte	Eonset (V)	$E_{1/2}$ vs RHE	Reference
CChK-Bp (Waste sugar beet pulp)	0.1M KOH	0.79	0.73	[51]
NCF900 (catkins)	0.1M KOH	0.82	0.66	[52]
Fe-N-C (wood)	0.1M KOH	0.98	0.90	[53]
Fe-N-C	0.1M KOH	/	0.39	[54]
Co-N-C	0.1M KOH	0.91	0.80	[55]
Fe-N-C (tea leaves waste)	0.1M KOH	0.93	0.84	[56]
Silicon self-doped biocarbon (sargassum spp.)	0.1M KOH	0.82	0.68	[57]
Fe–N/C (wheat stalk)	0.1M KOH	/	0.85	[58]
Fe5-PANI/C-MCS (corn stalks)	0.1M KOH	1.09	0.85	[59]
C-N-Fe-800	0.1M KOH	0.92	0.79	This work

References

[49] Bhattacharya, S.; Roy, S.; Barman, K.; Pramanik, A.; Das, A.; Lahiri, G. K.: Ruthenium containing molecular electrocatalyst on glassy carbon for electrochemical water splitting. *Dalton Trans.* **2022**, 51 (20), 7957–7965.

[50] Lee, S. H.; Sullivan, I.; Larson, D. M.; Liu, G.; Toma, F. M.; Xiang, C.; Drisdell, W. S.: Correlating oxidation state and surface area to activity from operando studies of copper CO electroreduction catalysts in a gas-fed device. *ACS Catal.* **2020**, 10 (14), 8000–8011.

[51] Chernysheva, D.V.; Konstantinov, M.S.; Sidash, E.A.; Klushin, V.A.; Tokarev, D.V.; Andreeva, V.E.; Butova, V.V.; Kaichev, V.V.; Smirnova, N.V.; Ananikov, V.P.: Tuning sugar biomass waste conversion for the preparation of carbon materials for supercapacitors and catalysts for oxygen reduction. Energy Technol. **2022**,11(3), 2201145.

[52] Liu, A.; Ma, M.; Zhang, X.Q.; Ming, J.; Jiang, L.; Li, Y.; Zhang, Y.J.; Liu, S.Q.: A biomass derived nitrogen doped carbon fibers as efficient catalysts for the oxygen reduction reaction. J. Electroanal. Chem. **2018**,824, 60-66.

[53] Li, D.D.; Han, Z.; Leng, K.Y.; Ma, S.H.; Wang, Y.; Bai, J.B.: Biomass wood-derived efficient Fe-N-C catalysts for oxygen reduction reaction. J. Mater. Sci. **2021**,56, 12764-12774.

[54] Li, R.N.; Li, X.W.; Liu, Q.C.: Effect of Fe-N-C single atom/cluster catalyst on ORR of PEMFC. Int. J. Hydrogen Energy. **2025**,139, 718-729.

[55] Zhu, Z.J.; Chen, C.M.; Cai, M.Q.: Porous Co-N-C ORR catalysts of high performance synthesized with ZIF-67 templates. Mater. Res. Bull. **2019**,114, 161-169.

[56] Yu, L.Q.; Wang, H.; Chen, S.L.; Wen, T.E.; Huang, B.C.; Jin, R.C.: Biomass derived Fe-N/C catalyst for efficiently catalyzing oxygen reduction reaction in both alkaline and neutral pH conditions. Chin. Chem. Lett. **2023**,34, 107236.

[57] González Zeferino, I.; Valenzuela-Muñiz, A.M.; Verde Gómez, Y.: Studies of the atomic rearrangement of carbon and silicon derived from Sargassum spp. and its effect on ORR activity. Int. J. Hydrogen Energy. **2025**,141, 1109-1117.

[58] Zhao, X.T.; Maimaitiyiming, X.; Sawut, A.; Kuerban, Z.: Fabrication of biocarbon catalyst for ORR by modulating the cellulose fraction of wheat stalk. Int. J. Hydrogen Energy. **2024**, 77, 1268-1275.

[59] Liu, J.M.; Maimaitiyiming, X.; Kuerban, Z.: Fe/N-doped porous carbon materials derived from corn stalks as ORR electrocatalysts for DMFC. Mater. Lett. **2024**,354, 135336.
